# Colorectal cancer in young adults: a retrospective study of 32 tunisian patients

**DOI:** 10.11604/pamj.2018.31.62.11043

**Published:** 2018-09-27

**Authors:** Faten Limaiem, Sonia Azzabi, Asma Sassi, Sabeh Mzabi, Saadia Bouraoui

**Affiliations:** 1 University of Tunis El Manar, Faculty of Medicine, Tunis, Tunisia

**Keywords:** Colorectal cancer, young adults, treatment

## Abstract

Young people under the age of 40 with colorectal cancer represent a distinct subgroup with a more aggressive disease behaviour compared to older patients. This study aim to provide an updated overview on clinicopathological features, treatment and outcome of colorectal cancer in young adults under the age of 40. In our retrospective study, we reviewed 32 cases of colorectal cancer in young adults aged less than 40 years that were diagnosed at the pathology department of Mongi Slim hospital over a fifteen-year period (April 2000 - November 2014). Our study group included 13 male and 19 female patients (sex-ratio M/F = 0,68) between 17 and 39 years of age (mean = 31,25 years). The presenting clinical symptoms were dominated by altered bowel habits (n=17), followed by bleeding per rectum (n=16). Histopathological examination of the surgical and biopsy specimens established the diagnosis of mucinous adenocarcinoma in nine cases, well-differentiated adenocarcinoma in 11 cases, moderately differentiated adenocarcinoma in six cases, poorly differentiated adenocarcinoma in four cases and signet ring cell carcinoma in two cases. The tumours were classified after surgery as stage I (n = 2) (6%), stage IIA (n = 7) (22%), stage IIB (n=4) (13%), stage IIC (n=1) (3%), stage IIIB (n=8) (25%), stage IIIC (n= 4) (12%), stage IVA (n=4) (13%) and stage IVB (n=2) (6%). During the follow-up period which ranged between one month and 9 years, local recurrence of the tumour occurred in six cases, seven patients had hepatic metastases and seven patients died after a mean follow-up period of seven months. Molecular genetic studies are increasing the understanding of the pathobiology of colorectal cancer and may ultimately allow at-risk patients to be identified at an earlier stage.

## Introduction

Colorectal cancer (CRC) arises predominantly in the older population, with more than 90% occurring after the age of 55 years [1]. Colorectal cancer is infrequent before 40 years of age and is associated with a poor outcome due to advanced stage at diagnosis and poor differentiation. Controversies and debates regarding the characteristics and the prognosis of CRC in the young population still exist. Hence, young patients represent a specific subgroup of patients requiring further survey. The aim of this study was to retrospectively review the presentation and outcomes of 32 young patients under 40 years of age with CRC over the past 15 years. Our results are analyzed in comparison to a review of the literature.

## Methods

We undertook a retrospective study of 32 young patients under 40 years of age who were operated on for colorectal cancer at the surgery department of Mongi Slim hospital of Tunis between April 2000 and November 2014. The cases were retrieved from the files of the registry of the surgery department of the same hospital. Medical records were scrutinized for epidemiologic characteristics, initial manifestations of the disease, methods of diagnosis, laboratory findings, surgical or palliative therapy and overall morbidity and mortality. Diagnosis of colorectal cancer was based upon histopathological findings. All patients underwent imaging evaluation during the preoperative period. All specimens were surgically obtained. Tissues were fixed in 10% phosphate buffered formaldehyde, embedded in paraffin and sections were prepared for routine light microscopy after staining with haematoxylin and eosin. Disease stage was determined according to the seventh edition of the TNM classification of the Union Internationale Contre le Cancer (UICC). Tumours were histologically classified according to the 2010 World Health Organisation Classification Of Tumours of the Digestive System. Patient confidentiality was maintained.

## Results

**Patient characteristics**: Patients under 40 years of age with CRC diagnosed between April 2000 and November 2014 represented 7,53% of all CRC (n=425). There were 13 male and 19 female patients (sex-ratio M/F = 0,68) between 17 and 39 years of age (mean = 31,25 years). Five patients presented with co-morbidities namely Crohn´s disease (n=2), duodenal ulcer (n=1), chronic gastritis (n=1) and celiac disease (n=1). Five patients had a family history of CRC, one patient had a family history of familial adenomatous polyposis and one patient had a family history of renal and bladder cancer.

**Presenting symptoms**: The presenting clinical symptoms were dominated by altered bowel habits (n=17), followed by bleeding per rectum (n=16), abdominal pain (n=15), altered general health (n=11), occlusive syndrome (n=8), rectal syndrome (n=6), abdominal mass (n=4), vomiting (n=1) and weight-loss (n=1).

**Biological tests**: Preoperative serum carcinoembryonic antigen levels were performed in 12 cases. They were elevated in four cases (> 5ng/ml) and within normal range in eight cases (< 5ng/ml). Preoperative serum carbohydrate antigen CA 19-9 levels were performed in three cases. They were elevated in one case ( > 37 U/ml) and within normal range in two cases ( < 37 U/ml).

**Figure 1:**
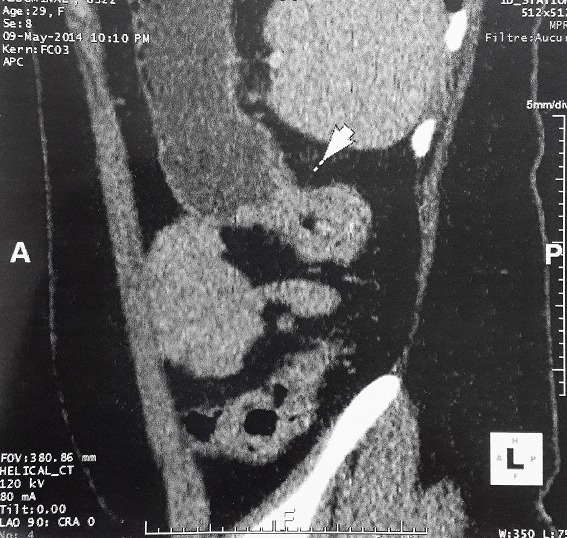
Abdominal CT scan showing irregular parietal thickening of the descending colon with important dilation of the colon upstream

**Figure 2:**
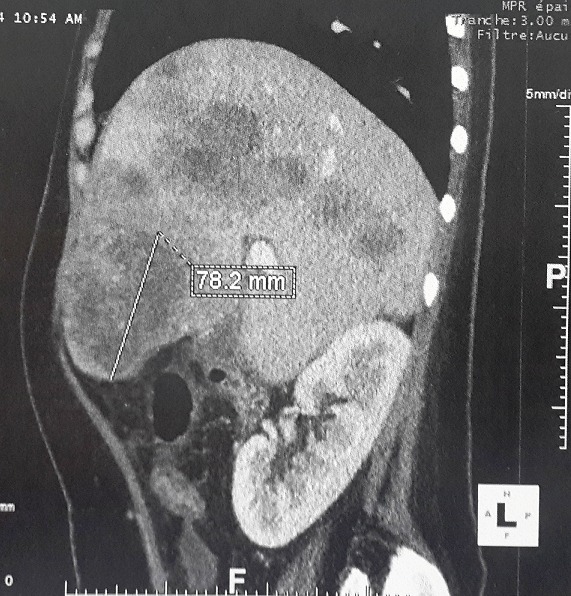
Abdominal CT scan demonstrating multiple enhancing hypodense lesions of the liver

**Localization of colorectal cancer, endoscopic and radiological findings (** **Figure 1** **):** The tumour localisation was classified in four groups: rectal cancers (41%, n = 13) included tumours within a distance of 16 cm or less from the anal verge, measured with a rigid sigmoidoscope. All tumours above 16 cm from the anal verge were declared as colon cancers (59%; n = 19) and subdivided in tumours of the sigmoid colon (n= 10), left colon (including descending colon (n=2), left flexure (n=3) and transverse colon (n=1)) and right colon (including right flexure, ascending colon (n=1), cecum (n=2) and appendix) according to the operation protocol. Colonoscopy and / or rectoscopy were performed in all cases. Postoperative computed tomography scan showed adrenal metastases in two cases, hepatic metastases in seven cases (Figure 2), peritoneal effusion in five cases and lung metastases in one case.

**Treatment strategies**: One patient had neoadjuvant chemotherapy and another received neoadjuvant chemoradiation. Thirty-one patients underwent surgical treatment including right hemicolectomy (n=4), left hemicolectomy (n=5), sigmoidectomy (n=4), total colectomy (n=2), anterior resection (n=13), abdominoperineal amputation (n = 2), total procto-colectomy (n=1). Postoperatively, 20 patients received adjuvant chemotherapy. One patient with peritoneal carcinomatosis discovered peroperatively had only palliative chemotherapy.

**Figure 3:**
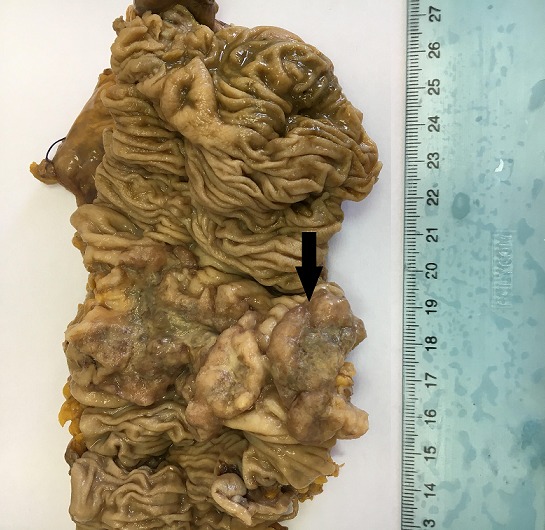
Gross findings of colonic adenocarcinoma: exophytic tumour with intraluminal growth (black arrow)

**Figure 4:**
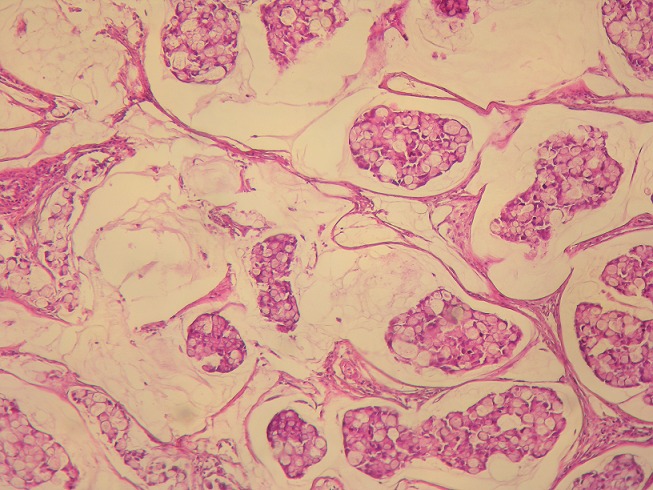
Histological findings of mucinous adenocarcinoma. Clusters of signet ring cells within pools of extracellular mucin, (Haematoxylin and eosin, magnification × 200)

**Pathologic findings**: Grossly, carcinomas of our series had variable macroscopic appearances : exophytic or fungating with predominantly intraluminal growth (n = 6) (Figure 3), exophytic and ulcerated (n = 14), endophytic and ulcerative with predominantly intramural growth (n = 11) (Figure 2). Associated colorectal polyps were identified in nine cases. For only one patient who received palliative chemotherapy without surgical treatment, we could not appreciate the gross appearance of the tumour. Histopathological examination of the surgical and biopsy specimens established the diagnosis of mucinous adenocarcinoma in nine cases (28%) (Figure 4), well-differentiated adenocarcinoma in 11 cases (34%), moderately differentiated adenocarcinoma in six cases (19%), poorly differentiated adenocarcinoma in four cases (13%) and signet ring cell carcinoma in two cases (6%). Perineural invasion was found in 15 cases (47%) and lymphovascular invasion in 10 cases (31%). The nine colorectal polyps corresponded histologically to: tubulovillous adenoma with an invasive degenerated focus (n=1), serrated adenoma (n=1), tubulovillous adenoma with high grade dysplasia (n=2), tubulovillous adenoma with low grade dysplasia (n=5).

**Staging of colorectal carcinoma according to UICC (7^th^edition)**: In our series, CRC were classified after surgery as: stage I (n = 2) (6%), stage IIA (n = 7) (22%), stage IIB (n=4) (13%), stage IIC (n=1) (3%), stage IIIB (n=8) (25%), stage IIIC (n= 4) (12%), stage IVA (n=4) (13%) and stage IVB (n=2) (6%).

**Operative morbidity and postoperative complications**: Postoperative course was uneventful in 17 cases. Postoperative complications (surgical and nonsurgical) occurred in 14 patients (44%).

**Surgical complications**: Colorectal anastomotic leakage (n=2) and rectovesical fistula (n=1).

**Non surgical complications**: Hemorrhagic shock (n=7), sepsis (n=1), bilateral pleural effusion (n=1), oligoanuria (n=1), and hypokalemia (n=1).

**Follow-up and evolution**: The mean follow-up period of our patients was 29 months (range: 1 month - 9 years). Local recurrence of the tumour occurred in six cases. In the control examination, CT scan revealed liver metastases in seven cases. Seven patients died after a mean follow-up period of 7 months (range: 1-13 months).

## Discussion

The current definition of young CRC patients remains controversial. Some studies used the cutoff age of 50 years, while others used 30 years or 45 years. But to date, majority of studies in the literature used the cutoff age of 40 years to denote a young patient with CRC. This lack of a standard definition makes it difficult to make meaningful comparisons between different studies. In our study, we defined young patients using an upper limit of 40 years as most studies reported. The Surveillance, Epidemiology and End Results (SEER) database estimates showed that the incidence of CRC in young adults has been increasing over the last 25 years, while the overall incidence has remained relatively stable for older adults [2]. It has been postulated that since routine CRC screening omits young adults, CRC cannot be prevented in this group by removing premalignant polyps and this may potentially explain a lack in the decrease in incidence [3]. A review of several studies on young (<40 years) CRC patients has shown that its frequency varies from 0,4% to 35,6% of all CRC patients, with an adjusted average of about 6% [4]. In our series, CRC in patients under 40 years of age represented 7, 53% of all CRC diagnosed during the same period. Literature review of series of CRC in young patients under 40 years is summarized in Table 1 [5-17].

**Table 1:** Colorectal cancer in patients under 40 years: literature review [5-17]

Authors	Year	Number of cases	Mean age (Range)	Sex-ratio (M/F)
Steffen B et al., [5]	1980	951	-	0,88
Ohman U et al., [6]	1982	48	(21 - 39)	0,92
Adolff M et al., [7]	1986	32	-	0,88
Smith C et al., [8]	1989	50	35 (7-39)	1,17
Pocard M et al., [9]	1997	80	34 (19-40)	1,35
Paraf F et al., [10]	2000	34	34,7+/-4,7	0,78
Sahraoui S et al., [11]	2000	88	31,4	0,9
Frizis H et al., [12]	2004	11	39 (37-40)	1,35
Fadlouallah M et al., [13]	2010	40	28 (17 - 40)	1,35
Ganapathi S et al., [14]	2011	59	-	0,96
Haroon N et al., [15]	2013	23	31 +/-5 (18-39)	1,55
Sudarshan V et al., [16]	2013	91	-	1,75
Domati F et al., [17]	2014	57	-	2,56
Our series	2016	32	31,25 (17-39)	0,68

The majority of CRC cases in young adults are sporadic in nature and the aetiology remains unclear. Several factors, however, have been proposed to contribute to the increasing rate of CRC in young adults, specifically unhealthy dietary choices and red meat consumption, physical inactivity and obesity [18, 19]. When a young adult is diagnosed with CRC, inherited high-risk CRC syndromes, such as familial adenomatous polyposis, Lynch syndrome, MUTYH-associated polyposis and the less common hamartomatous polyposis syndromes, must be considered. It is currently estimated that approximately 10 % of young-onset CRC is attributable to inherited syndromes; however, this has not yet been comprehensively studied. In our series, five patients had a family history of CRC and one patient had a family history of familial adenomatous polyposis. O'Connell *et al*. published a detailed systematic review of 55 articles pertaining to CRC in adults less than 40 years old in 2004 [4]. They found that of the 5051 individuals with CRC in their analysis, 51,4 % were men and 48, 6 % were women [4]. In our series, there was a female predominance with a sex-ratio M/F = 0, 68. The location of CRC was predominately left-sided: 54 % of individuals had CRC in the rectum and sigmoid colon, while 22 % had tumours in the ascending colon (cecum, ascending colon, and hepatic flexure), 11 % in the transverse and 13 % in the descending colon (splenic flexure, descending colon). In our series, 72% of patients had CRC in the rectum and sigmoid colon, while 9% had tumours in the ascending colon, 3% in the transverse and 16% in the descending colon. Results of previous investigations regarding young patients with CRC reported more advanced stages of disease, a worse survival and a more proximal localization of the tumour in the colon in younger than in older patients [20-23]. The common clinical presenting features of young patients with CRC are bleeding per rectum, altered bowel habit, anemia and weight-loss. In our series, the presenting clinical symptoms were dominated by transit disorders (n=17), followed by rectal bleeding (n=16), abdominal pain (n=15), altered general health (n=11), occlusive syndrome (n=8), rectal syndrome (n=6), abdominal mass (n=4), vomiting (n=1) and weight-loss (n=1). In a recent review of the SEER data of 11071 adolescents and young adults, over 72% presented with either regional or metastatic disease. In comparison, 50-58% of the general population presented with regional or metastatic disease [24]. Many authors postulate that a more biologically aggressive tumour occurs in young patients, based on the finding of a higher percentage of poorly differentiated and mucin-producing cancer [8, 25]. In one review of the SEER data, mucinous tumours constituted an average of 21% of the lesions found in young patients compared to 10-12% in older adults, while the percentage of tumours found to be poorly differentiated was 27% compared with 15% for adults over 40 years of age [4].

In our study, the mucinous tumours constituted 28% of young CRC and 13% of tumours were found to have poor differentiation. Previous studies have shown the presence of signet ring carcinoma in 1,7- 11,1% of young CRC [4]. In our study, they represented 6% of all CRC. On the other hand, there are recent population-based studies showing that young patients have not a significantly worse survival rate compared with older ones [4, 7, 26, 27]. In a recent review of the SEER data of 11071 adolescents and young adults the disease-specific survival and the overall survival were comparable to those of the general population [24]. On multivariate analysis, disease stage at the time of the diagnosis was the strongest predictor of mortality [24]. After controlling for disease stage, male gender, black race, and higher grade tumours were associated with worse survival [24]. In our series, local recurrence of the tumour occurred in six cases (19%). Seven patients had hepatic metastases (22%) and seven patients (22%) died after a mean follow-up period of 7 months. A genetic basis for tumorigenesis has been implicated in early onset CRC among young patients. Microsatellite instability has been identified in most of the patients with early onset of CRC, suggesting genetic etiology [28].

## Conclusion

In summary, this retrospective study from Tunisia provides an overview on clinical symptoms, radiological features, treatment and outcome in 32 patients under 40 years of age with CRC. Outcome of younger patients could be improved if screening colonoscopy would start at 40 years as young patients could be diagnosed at an earlier tumour stage. The early detection of CRC followed by a sufficient oncologic treatment is crucial regardless of age. Molecular genetic studies are increasing the understanding of the pathobiology of colorectal cancer and may ultimately allow at-risk patients to be identified at an earlier stage. Future interventions tailored to this young population may help achieve improvements in their overall prognosis.

### What is known about this topic

- Colorectal cancer is infrequent before 40 years of age and is associated with a poor outcome due to advanced stage at diagnosis, delayed diagnosis and poor differentiation;
- Accurate diagnosis and staging of colorectal cancer is critical for optimal treatment planning and for determining prognosis.

### What this study adds

- This is the first retrospective study from Tunisia that provides an overview on clinical symptoms, radiological features, treatment and outcome in 32 young Tunisian patients under 40 years of age with colorectal cancer.

## Competing interests

The authors declare no competing interests.
